# Screening for latent tuberculosis infection among undocumented immigrants in Swiss healthcare centres; a descriptive exploratory study

**DOI:** 10.1186/1471-2334-9-34

**Published:** 2009-03-24

**Authors:** Patrick Bodenmann, Paul Vaucher, Hans Wolff, Bernard Favrat, Fanny de Tribolet, Eric Masserey, Jean-Pierre Zellweger

**Affiliations:** 1 Department of Ambulatory Care and Community Medicine, University of Lausanne, Switzerland; 2 Department of Community Medicine, Geneva University Hospital, Switzerland; 3 Point d'Eau, Vulnerable Population Urban Healthcare Centre, Lausanne, Switzerland; 4 Department of Public Health, Canton of Vaud, Switzerland

## Abstract

**Background:**

Migration is one of the major causes of tuberculosis in developed countries. Undocumented patients are usually not screened at the border and are not covered by a health insurance increasing their risk of developing the disease unnoticed. Urban health centres could help identify this population at risk. The objective of this study is to assess the prevalence of latent tuberculosis infection (LTBI) and adherence to preventive treatment in a population of undocumented immigrant patients.

**Methods:**

All consecutive undocumented patients that visited two urban healthcare centres for vulnerable populations in Lausanne, Switzerland for the first time were offered tuberculosis screening with an interferon-γ assay. Preventive treatment was offered if indicated. Adherence to treatment was evaluated monthly over a nine month period.

**Results:**

Of the 161 participants, 131 (81.4%) agreed to screening and 125 had complete examinations. Twenty-four of the 125 patients (19.2%; CI95% 12.7;27.2) had positive interferon-γ assay results, two of which had active tuberculosis. Only five patients with LTBI completed full preventive treatments. Five others initiated the treatment but did not follow through.

**Conclusion:**

Screening for tuberculosis infection in this hard-to-reach population is feasible in dedicated urban clinics, and the prevalence of LTBI is high in this vulnerable population. However, the low adherence to treatment is an important public health concern, and new strategies are needed to address this problem.

## Background

Each year, more than 8 million people develop active tuberculosis (TB) worldwide with regional variability. It is the most common cause of death from communicable diseases [1,2]. Movement of people between countries has a large influence on the incidence of TB in Western-Europe [3-5]. Several studies have shown that most reported cases are due to reactivation of infections acquired abroad but that transmission to other people in the host country is rare [6-9]. Therefore, screening for tuberculosis among immigrants is performed in many developed countries with the aim of detecting and treating the active disease [10,11] or a latent tuberculosis infection (LTBI) [12-14]. The introduction of interferon-γ assays has improved specificity for detecting LTBI compared with the tuberculin skin test [15,16]. This promises a considerable improvement in the cost-effectiveness of targeted screening programs [17-20]. However, there is no clear consensus regarding the true benefits of screening and it is difficult to assess treatment policies [21].

In practice, screening policies address immigrants that enter into the country via an official channel (asylum claim & working permit). In Switzerland, a screening policy exists for asylum seekers and refugees at the border, but no system exists for undocumented immigrants from countries with a high prevalence of tuberculosis [22,23]. The latter represent a large proportion of the foreign-born population (estimates vary between 150,000 and 300,000 people for a total immigrant population of 7.5 million) [24]. Targeted screening programs may be cost-effective [25], but further studies are needed to evaluate the prevalence of LTBI, the acceptability of screening, and adherence to treatment in this hard-to-reach vulnerable population.

## Methods

We conducted a pilot trial to investigate the acceptance, prevalence of positive findings, and adherence to treatment of undocumented immigrant patients offered LTBI screening. We offered a free tuberculosis screening procedure to all consecutive, undocumented immigrants that were over 15 years old, had no major psychiatric disabilities, and were visiting one of two low-threshold health-care premises for the first time in Lausanne. The recruitment proceeded during a six-month period in 2007 at the Department of Ambulatory Care and Community Medicine of the University Hospital and at the Point d'Eau, Vulnerable population urban healthcare centre. Patients were evaluated by nurse practitioners or primary care physicians trained in community medicine.

Patients were asked to answer a health questionnaire in their preferred language that highlighted symptoms or history that might be associated with tuberculosis (cough, sputum, night sweating, weight loss, prior contact with tuberculosis, prior treatment for tuberculosis, and smoking). From each patient, 8 ml of blood was collected in a Vacutainer-CPT. Within the next 24 h, an enzyme-linked immunospot γ-interferon assay (T-Spot.TB™, Oxford Immunotec) was performed to assess the previous tuberculosis infection status. This test has been shown to have a sensitivity of 90% and a specificity of 93% to detect latent tuberculosis [26]. If results were inconclusive, the assay was repeated. Patients with suspect symptoms, a prior history of tuberculosis, prior contact with tuberculosis, or a positive interferon-γ test result had a chest X-ray and a medical examination by a physician. A bacteriological examination of sputum was performed in all patients that had suspected symptoms or an abnormal X-ray. Patients with active tuberculosis were treated in our Department according to the current Swiss Guidelines [27,28]. Patients that had a positive interferon-γ assay with no signs of tuberculosis were offered preventive treatment. Treated patients were followed for a six-month period. We monitored the acceptance of screening, the number of patients that returned for test results, the number of patients that accepted treatment, and the number of patients that finished treatment.

Sample size was calculated to assure a 0.1 margin of error for the LTBI prevalence rate with a significant level set at 95% and an expected prevalence of 0.5 (worse case scenario). We estimated that 97 patients were necessary to sufficiently power the analysis. In anticipation of loss to follow-up and missing data, we included 130 participants in the study. Prevalence and acceptance rates are given with a 95% confidence interval. Other results are given in a descriptive form without inference, as we expected small sample sizes.

All patients provided written informed consent for participation, the protocol was approved by the Lausanne University Ethical Committee (protocol 183/06), and the study conformed to the standards defined in the Declaration of Helsinki.

## Results

**Figure 1:**
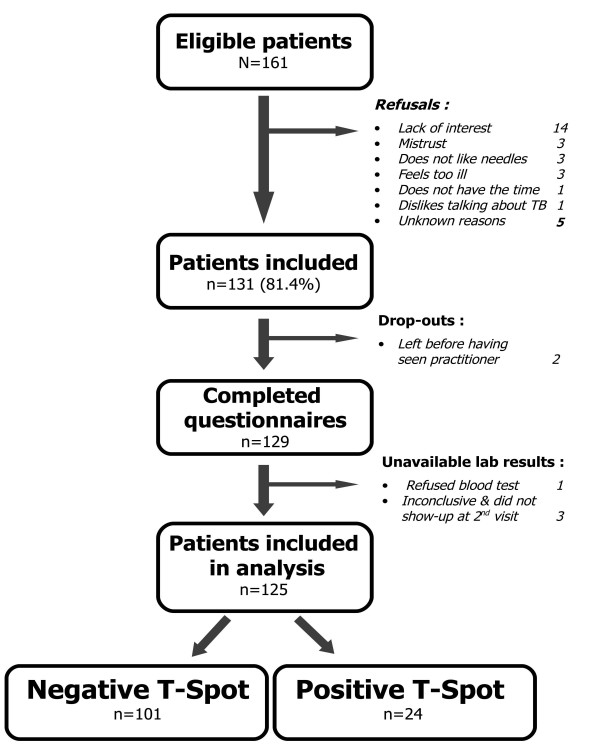
**Flow-chart of patient inclusion in the study**.

Between January and July 2007, 161 undocumented immigrants that visited one of two medical centres for the first time were asked to participate in this study. Among these, 131 agreed to answer a questionnaire regarding symptoms and history associated with tuberculosis and had a blood sample drawn for an interferon-γ assay. The questionnaire acceptance rate was 81.4% (CI95% 74.5;87.1). However, of these 131 patients, two patients left the waiting room before seeing the practitioner, and one refused to give a blood sample. Thus, 128 laboratory samples were tested, and five of these were inconclusive. Twenty-five patients (19.5%) did not return for their test results, and three of these had an initial inconclusive laboratory result. Confirmation of the inconclusive essay revealed on positive and one negative patient. Laboratory results were therefore available for 125 patients (Figure 1).

The demographic data are given in Table 1. The mean age of the participants was 34.8 years, 47.2% were female, and 64% were single. Most of the patients (83.2%) entered Switzerland without receiving the official tuberculosis screening procedure for asylum seekers. The majority of patients were undocumented immigrants born in Latin America (51.2%), and many were from Sub-Saharan Africa (19.2%). Most of the patients (76.8%) were from countries that had a high incidence rate of active tuberculosis (≥ 50/100,000 per year). The mean length of time spent in Switzerland was under two years for 52.8% of the population. Only five out of the 24 participants that tested positive and 15 of the 101 participants that tested negative for TB reported previous contact with a person that had TB Thus, the sensitivity of this question was 20.8% and the specificity was 85.1%.

**Table 1:** Population characteristics

Population characteristic	Interferon-γ assay		Total
	Positive n = 24 n *(%)*	Negative n = 101 n *(%)*	n = 125 *n (%)*
**Age**			
<20	0 *(0)*	3 *(3.0)*	3 *(2.4)*
20–39	13 *(54)*	78 *(77)*	91 *(72.8)*
40–59	11 *(46)*	18 *(18))*	29 *(23.2)*
60 or more	0 *(0)*	2 *(2)*	2 *(1.6)*
			
**Gender**			
Male	13 *(54)*	53 *(52)*	66 *(52.8)*
			
**Level of education**			
Compulsory or less	11 *(46)*	46 (46)	55 *(45.6)*
			
**Reasons for migrating**			
Economical	16 *(64)*	63 *(63)*	79 *(63.2)*
Political persecution	2 *(8)*	19 *(19)*	21 *(16.8)*
Religious persecution	1 *(4)*	2 *(2)*	3 *(2.4)*
Disease	4 *(17)*	2 *(2)*	6 *(4.8)*
Family grouping	4 *(17)*	16 *(16)*	20 *(16)*
Studies	2 *(8)*	8 *(8)*	10 *(8.0)*
Other	1 *(4)*	6 *(6)*	7 *(5.6)*
			
**Arrived in Switzerland**			
Less than 2 years	12 *(48)*	54 *(54)*	66 *(52.8)*
2–7 years	6 *(25)*	38 *(38)*	44 *(35.2)*
More than 7 years	6 *(24)*	9 *(9)*	15 *(12.0)*
			
**Tobacco**			
Smoker	9 *(37)*	39 *(39)*	48 *(38.4)*
			
**Close contact with TBC**			
Reported	5 *(21)*	15 *(15)*	20 *(16.0)*
Not reported	19 *(79)*	86 *(85)*	20 *(84.0)*
			
**Proximity of at least one hour with someone during previous 3 months**			
Disco or dancing	8 *(33)*	43 *(43)*	51 *(40.8)*
Religious meetings	10 *(42)*	41 *(41)*	51 *(40.8)*
Interior sports	1 *(4)*	22 *(22)*	23 *(18.4)*
Restaurants or pubs	4 *(17)*	16 *(16)*	20 *(16)*
Welfare hostel	1 *(4)*	5 *(4)*	6 *(4.8)*
Other	1 *(4)*	8 *(8)*	9 *(7.2)*

The interferon-γ assay indicated that 24 immigrants were positive for TB (19.2%) [CI 95% 12.7–27.2]. Of these, two had smear-positive tuberculosis (1.6%) [CI95% 0.2–5.7], three had a history of prior treatment for tuberculosis, one had a history of prior treatment for LTBI, and 18 had no active or prior tuberculosis (14.4%, CI95% 8.8–21.8). One patient was suspected to falsely have a negative interferon-γ assays as he had been treated for a sputum confirmed active tuberculosis two years previously.

**Figure 2:**
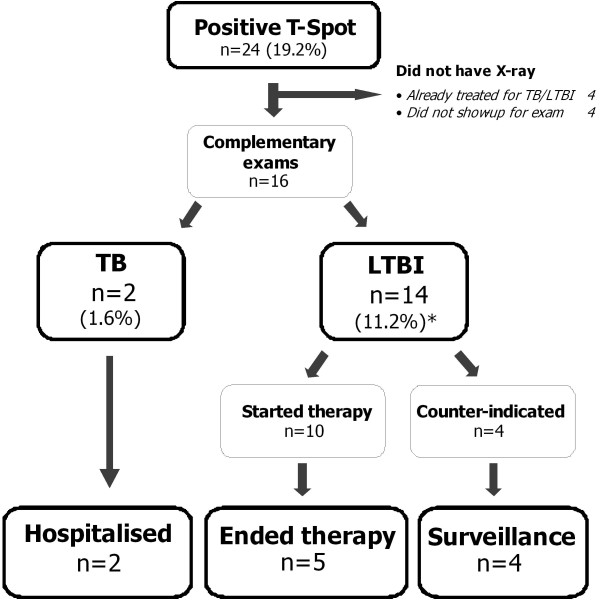
**Adherence to therapy from testing to the end of prophylactic therapies**. * Total number of participants with LTBI was expected to be 18. We assumed that the four patients that did not show up for further examination did not have active TB.

The two patients with active tuberculosis were isolated and treated according to the current Swiss Guidelines [27]. Both were adherent to treatment and were cured. Among the 14 immigrants with no prior history of TB and a positive interferon-γ assay result, four had counter-indications for preventive treatment (high age, liver disease), four did not show up for further examinations, and 10 accepted preventive treatment for LTBI. Of the 10 that accepted preventative treatment, only five followed the treatment until the scheduled completion. Therefore, full treatment was sustained by 5/18 patients with LTBI (Figure 2).

## Discussion

In our study, 19.2% (24/125) of the undocumented immigrant patients had positive interferon-γ assay results and 14.4% (18/125) had LTBI that was previously undetected. Carvalho et al [25] found similar results (15%; 15/100) for undocumented immigrant patients attending an Italian health care service. Although the sample sizes were small in both these studies, the observed prevalence of positive interferon-γ results appeared to be substantial compared to other population groups, including: German subjects that had been in close contact with an index case (10%) [29], healthcare workers in Denmark (1.4%)[30] or Japan (9.9%)[31], American Navy recruits (0.6%)[32], and the general Japanese population (7.1%)[33]. Prior exposure to an index case with pulmonary TB in the country of origin appeared to be the primary cause of LTBI in low-incidence rate countries [3]. Furthermore, the risk of reactivating a LTBI into active tuberculosis seemed to mainly be related to the initial exposure in high incidence countries of origin and not to other risk behaviours or environmental conditions in the host country [7,34]. More than 3/4 of undocumented immigrants consulting healthcare centres in Western Switzerland are from countries with incidence rates of tuberculosis above 50 cases/100,000 people per year. This is most likely the major reason for the high prevalence of LTBI in this population.

Targeted tuberculosis screening seems to be more cost-effective for preventing outbreaks than systematic screening [17,21]. Clinical trials have shown that preventive treatment reduced infection rates by more than half when strict adherence was achieved [14,35]. Most cost-effectiveness studies [18-20,36,37] are based on estimates that do not take into consideration the number of positive interferon-γ assays that are due to past infections where treatment was interrupted. Nevertheless, our study showed a potential benefit of screening and offering preventive therapy for this specific vulnerable population. Preventing the emergence of new index cases in this hard-to-reach population that often lives in close quarters could substantially reduce expenses related to controlling an outbreak [38]. The high prevalence of LTBI suggests that screening with an interferon-γ assay could be cost-effective under conditions that promoted adherence to the end of therapy for most patients [36]. However, the risk of developing tuberculosis is much higher for individuals with positive intereron-γ results than those with a positive tuberculosis skin test [39].

The acceptance rate for screening was high (81.4%). Using nurse practitioners and trained primary physicians in secure environments could help build the trust of this hard-to-reach population. Maintaining confidentiality could be essential to assure trust [40].

On the other hand, there are obstacles in ensuring that patients follow their treatment to the end. Only five patients out of eighteen with LTBI completed the preventive therapy in our study. Ailinger et al [41] observed an adherence prevalence of 72% for Latino patients that were undocumented immigrants in Washington DC. A prior study from our institution observed that the rate of adherence was 76% in a group composed mainly of foreign workers and refugees [42]. Therefore, adherence to treatment may depend on the setting and the confidence the patients have in the system (fear of denunciation and rejection). Supervising [43] or monitoring [44] drug administration, offering free access to care [45], using models of explanation based on the patient's representation of his own health [46], and relying on trained nurse practitioners for screening [47,48] could improve compliance. Furthermore, compared to tuberculosis skin tests, the introduction of interferon-γ assays has largely improved the specificity of LTBI detection; thus, the number of patients that would receive preventive treatment without needing it can be reduced. This knowledge might raise the motivation of healthcare workers in their efforts to follow patients and help them adhere to the proposed treatment.

Our study has several weaknesses. The small size of the population sample led to a lack of precision in the results. The absence of a non-migrant control group made it difficult to compare the prevalence of LTBI in migrants with that of the local population; however, we considered it safe to assume that the local population living in Switzerland had a much lower prevalence of LTBI than the study population. The setting of our study could limit the generalisability of our results to rural migrants attending a healthcare centre. Patients who do not attend a healthcare centre could be younger and less deprived than those included in the study. Our population could be more at risk to have been in contact with tuberculosis. Our results are therefore limited to quantifying the prevalence of latent tuberculosis in a setting in which it is possible to consider targeted screening and prevention programs to take place. Finally, interferon-γ positivity may not equate perfectly with latent TB infection. As such additionally data using another interferon-γ or the tuberculosis skin test would have been helpful.

## Conclusion

Our study shows the benefit of using highly sensitive assays for detecting LTBI on targeted populations at risk that easily accept screening in appropriate health settings by trained nurse practitioners and primary care physicians. Nevertheless, methods for improving adherence to treatment are lacking. Our results demonstrate the high prevalence of LTBI in migrants and emphasize the need for developed countries to invest in programs that reduce the transmission of tuberculosis worldwide; in particular, the focus should be on undocumented migrants that frequently come from countries with high incidence rates of tuberculosis.

## Abbreviations

LTBI: latent tuberculosis infection; TB: active tuberculosis; CI: confidence interval; HIV: human immunodeficiency virus.

## Competing interests

The authors declare that they have no competing interests.

## Authors' contributions

PB has made substantive contribution to conception, acquisition of data, design and interpretation of data. PV has contributed to conception, design, analysis and interpretation of data. PB and PV have drafted the manuscript. HW, BF and EM have contributed to the interpretation of data and critically revised the manuscript. FdT has contributed to acquisition of data and revising the manuscript. JPZ has made contribution to conception and design, interpretation of data and has revised the manuscript with important intellectual content. All authors have given their approval for this version to be published.

## Pre-publication history

The pre-publication history for this paper can be accessed here:

http://www.biomedcentral.com/1471-2334/9/34/prepub
